# A Weibull distribution accrual failure detector for cloud computing

**DOI:** 10.1371/journal.pone.0173666

**Published:** 2017-03-09

**Authors:** Jiaxi Liu, Zhibo Wu, Jin Wu, Jian Dong, Yao Zhao, Dongxin Wen

**Affiliations:** School of Computer Science and Technology, Harbin Institute of Technology, Harbin, Heilongjiang Province, China; West Virginia University, UNITED STATES

## Abstract

Failure detectors are used to build high availability distributed systems as the fundamental component. To meet the requirement of a complicated large-scale distributed system, accrual failure detectors that can adapt to multiple applications have been studied extensively. However, several implementations of accrual failure detectors do not adapt well to the cloud service environment. To solve this problem, a new accrual failure detector based on Weibull Distribution, called the Weibull Distribution Failure Detector, has been proposed specifically for cloud computing. It can adapt to the dynamic and unexpected network conditions in cloud computing. The performance of the Weibull Distribution Failure Detector is evaluated and compared based on public classical experiment data and cloud computing experiment data. The results show that the Weibull Distribution Failure Detector has better performance in terms of speed and accuracy in unstable scenarios, especially in cloud computing.

## Introduction

Cloud computing has become a new computing model that provides elastic, on-demand and robust services [1]. Services in cloud computing may be virtualized with specific servers that host abstracted details [2]. Many legacy applications are being migrated to the cloud computing platform [3–4]. In cloud computing, the cloud service environment can be dynamic and unexpected because servers may be active, busy, offline or may have even crashed for various reasons [5]. It is important to address the variability and provide an effective control scheme to guide service conditions and cloud resources (such as energy and throughput management [6–7]). Fault tolerance schemes are designed to provide reliable and continuous services in cloud computing despite the failures of some servers [8–10]. As an essential building block for cloud computing, a failure detector (FD) plays a key role in the engineering of such dependable systems [11]. Effective failure detection can detect failures in a timely and accurate way. In cloud computing, the FD adapts to the various network conditions. Moreover, it is necessary to satisfy different quality of service (QoS) requirements of multiple cloud applications simultaneously [12].

The accrual FD, proposed by Defago [13], provides a flexible mechanism for failure detection in large-scale distributed systems. It allows a decoupling between the monitoring and interpretation of traditional FDs and outputs a continuous value associated with time to represent the suspicion level of the detected process rather than binary results (trust or suspect). Thus, this FD is suitable for deployment in cloud computing. In accrual FD, it is necessary to compute the suspicion level based on the distribution of past heartbeat inter-arrival times. In [14–15], the heartbeat inter-arrival time follows a normal and exponential distribution, respectively. However, the cloud service environment is dynamic and unexpected; such a situation was exacerbated when mobile cloud computing emerged. The existing distribution assumptions of heartbeat inter-arrival time are not reasonable for the cloud computing scenario. To find a reasonable distribution assumption, we select two groups of actual data (from WAN and the cloud computing platform) to analyze the distribution of heartbeat inter-arrival time. The results show that the Weibull distribution is a more reasonable distribution assumption for heartbeat inter-arrival time in cloud computing.

Therefore, an accrual FD based on the Weibull distribution is proposed, called Weibull Distribution Failure Detector (WD-FD). In WD-FD, a sliding window is used to maintain the most recent samples of the arrival time. These samples are used to estimate the parameters of the Weibull distribution. With such situation information, the suspicion level *w*_*d*_ is calculated to match the recent network condition. WD-FD can adapt well to the dynamic and unstable network conditions in cloud computing. The QoS of our algorithm has been evaluated and compared to the existing failure detection algorithms in terms of mistake rate, detection time and query accuracy probability. The experimental results demonstrate that WD-FD has better performance than other FDs in cloud computing.

The rest of this paper is organized as follows. In section ii, the related work of failure detectors is introduced. Section iii introduces the system model and presents the implementation of WD-FD. Section iv presents experiments on WAN and cloud computing. Finally, the work is concluded in section v.

## Related work

In this section, we first introduce the failure detection QoS metrics and then present the several existing main accrual FDs.

### Failure detection QoS metrics

A FD provides an information list of suspects due to which processes have crashed [16]. FDs are used in a wide variety of fields, such as grid computing [17], cluster management [12], peer-to-peer networks, and cloud computing [18]. In practice, many applications require some timing constraint on the behaviors of FDs. It is not acceptable that a process is suspected hours after it has crashed or the FD outputs several false positives. To solve this problem, Chen [19] proposed a series of metrics to specify the QoS of FD: how fast it detects actual failures and how well it avoids false detections. These metrics can quantitatively represent the detection speed and accuracy. We use *T* or *S* to represent whether a process is trusted or suspected. *T*-transition means that the output of the detector changes from *S* to *T*, while *S*-transition means that the output of the detector changes from *T* to *S*. The following three primary metrics are used to describe the QoS of a FD.

Detection time (*T*_*D*_) is the time that elapses from the moment when a process crashes to the time when it starts being suspected, i.e., when the final *S*-transition occurs.

Mistake rate (*λ*_*m*_) is the number of mistakes that a FD makes per unit time, i.e., it represents how frequently a FD makes mistakes.

Query accuracy probability (*P*_*A*_) is the probability that the output of a FD is correct at a random time.

**Fig 1:**
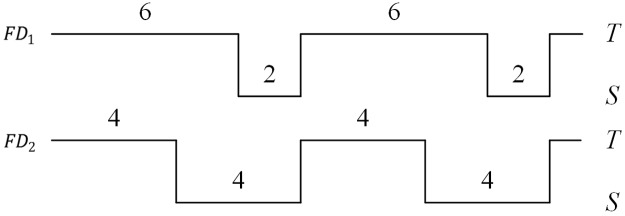
Query accuracy probability and mistake rate.

The first metric is related to a failure detector’s speed, while the remaining relate to its accuracy. In many cases, the mistake rate is not sufficient to describe the accuracy of a FD; simultaneously, the query accuracy probability is also needed. For example, Fig 1 shows that both FD_1_ and FD_2_ are detecting the process *p*. The two FDs have the same mistake rate (0.125) but different query accuracy probabilities (0.75 and 0.5).

### Accrual failure detector

Defago proposed a flexible mechanism for failure detection in a large-scale system, i.e., an accrual failure detector, which can adaptively satisfy various QoS requirements of different applications. It outputs a continuous value (*sl*_*qp*_(*t*)) to represent the suspicion level of the monitored process instead of providing information regarding a conventional binary nature (trust or suspect). An accrual FD will belong to the class *◇P*_*ac*_ if it satisfies the following properties:

Accruement: if process *p* is faulty, then eventually the suspicion level *sl*_*qp*_(*t*) monotonously increases at a positive rate.
Upper bound: if process *p* is correct, then the suspicion level *sl*_*qp*_(*t*) is bounded.

The *φ* FD [14] is the first implementation of accrual FD. It uses the heartbeat detection strategy as the basic detection strategy. Furthermore, it assumes that the heartbeat inter-arrival time follows a normal distribution. Therefore, the value of *φ* can be calculated as follows:

$$\varphi (T_{now})=-{\text{log}}_{10}(P_{later}(T_{now}-T_{last}))$$ (1)

where *T*_*last*_ is the time when the most recent heartbeat is received, *T*_*now*_ is the current time, and *P*_*later*_(*t*) is the probability a heartbeat will arrive more than *t* time units later than the previous one. According to the assumption of heartbeat inter-arrival time, *P*_*later*_(*t*) is given by the following equation:

$$P_{later}(t)=\frac{1}{\sigma \sqrt{2\pi }}\int_{t}^{\infty }e^{\frac{{\left(x-\mu \right)}^{2}}{2\sigma^{2}}}dx=1-F(t)$$ (2)

where *F*(*t*) is the cumulative distribution function of a normal distribution with *μ* and variance *σ*^2^. When the applications query the *φ* FD at time *T*_*now*_, *φ* FD will return a value *φ* to them. Then, each application compares the value of *φ* with its threshold Φ, which is given by different QoS requirements of multiple applications simultaneously.

The ED FD is similar to the *φ* FD. The difference is in the distribution assumption of heartbeat inter-arrival time. ED FD assumes that the heartbeat inter-arrival time follows an exponential distribution. Consequently, the suspicion level is given by a value, called *e*_*d*_, which is calculated as follows:

$$e_{d}=F(T_{now}-T_{last})$$ (3)

$$F(t)=1-e^{-\frac{t}{\mu }}$$ (4)

where *T*_*now*_, *T*_*last*_ and *μ* have the same meaning as for the *φ* FD. For this FD, the threshold is called *E*_*d*_.

In this section, the related work in the area of failure detection for distribution systems has been introduced. The implementations of accrual FD, i.e., *φ* FD and ED FD, are suitable for large-scale distributed systems. However, with the emergence of cloud computing, these FDs do not adequately comply with the new network conditions.

## An accrual failure detector based on Weibull distribution

In this section, the system model is firstly introduced. Second, two groups of data from different network conditions (WAN and cloud computing) are analyzed. Third, the implementation of WD-FD is described precisely. Finally, the correctness of the WD-FD algorithm is proven.

### System model

We consider a partially synchronous system consisting of a finite set of processes Π = {*P*_1_, *P*_2_, …, *P*_*n*_}. Each process behaves correctly until it crashes and is unable to recover. Any two processes can be connected by an unreliable communication channel. Because most FDs are implemented using the UDP protocol, we assume that the communication channel between processes is a fair-lossy channel [20], i.e., no message can be copied or modified and no new message can be created, and if a process *p* continues sending a message *m* to *q*, *q* will eventually receive *m*.

We assume the existence of some global time (unbeknownst to processes), denoted as global stabilized time (GST), and that processes always make progress; furthermore, at least *δ* > 0 time units elapse between consecutive steps (the purpose of the latter is to exclude the case where processes require an infinite number of steps in finite time).

To simplify the description, consider a system that consists of only two processes *p* and *q*, where *q* is monitoring *p*. Process *p* sends a message to *q* every Δ*t* time (sending interval) or is subject to crashing. Process *q* suspects process *p* if it does not receive any heartbeat message from *p* for a period of time determined by the freshpoint.

### Analysis of heartbeat inter-arrival time

To find a reasonable distribution assumption of heartbeat inter-arrival time, two groups of data from different platforms of WAN and cloud computing are analyzed. One group of data from WAN is classical experimental data [21] that has been used with *φ* FD [14]. The experiment exceeded one week, and more than 5 million samples were received. The average inter-arrival time of received samples is 103.9 ms, with a standard deviation of approximately 104.1 ms. The other group of data from cloud computing was collected by renting the Amazon EC2. The experiment lasted for 3 months, and 3 million samples were received. The average inter-arrival time of received samples is 2.12 s, with a standard deviation of approximately 0.1032 s.

**Fig 2:**
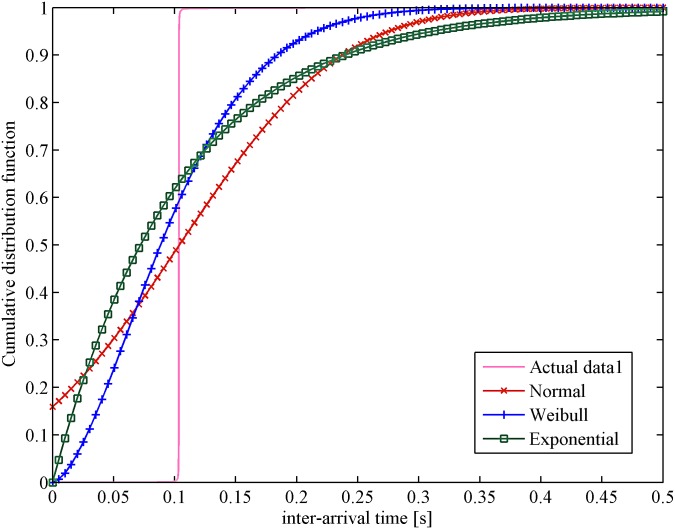
Probability distribution vs. inter-arrival time in WAN.

**Fig 3:**
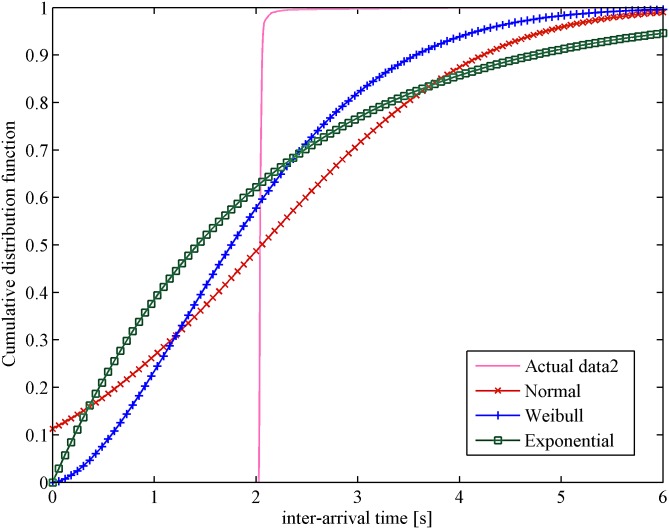
Probability distribution vs. inter-arrival time in cloud computing.

The cumulative distribution function of data from WAN is primarily analyzed by using the *dfittool* toolbar in MATLAB. The confidence level is set to 95%; then, three classical distributions (normal, exponential and Weibull distribution) are applied to fit the data. In Fig 2, the curve shows that the Weibull distribution is the nearest to the distribution of actual data. A similar result is obtained in the cloud computing experimental platform, as shown in Fig 3. According to these outcomes, it is clear that the Weibull distribution is the closest to real figures. Therefore, the Weibull distribution is a more reasonable assumption for the approximation of heartbeat inter-arrival time under unstable network conditions.

### Weibull distribution failure detector

Based on the above analysis of heartbeat inter-arrival time, we can assume that the heartbeat inter-arrival time follows the Weibull distribution. Therefore, the suspicion level of an accrual FD can be calculated as follows:

$$w_{d}(T_{now})=F(T_{now}-T_{last})$$ (5)

where *F*(*t*) is a Weibull distribution function and one has

$$R(t)=1-F(t)=e^{-{\left(t/\alpha \right)}^{\beta }}$$ (6)

when *t* > 0, and the parameters *α* and *β* can be computed via the least square method, which is described as follows.

According to Eq (6), the corresponding reliability function is

$$R(t)=1-F(t)=e^{-\alpha t^{\beta }}$$ (7)

To achieve a Weibull distribution transformation, we can consider the following equations:

y=ln(−ln(1−F(t))) (8)

x=lnt (9)

Thus, Eq (7) is eventually converted to

y=y(x)=β(x−lnα) (10)

For the samples of heartbeat inter-arrival time (*t*_1_, *t*_2_, ⋯, *t*_*n*_) in the sliding window, the value of *R*(*t*_*i*_) (*i* = 1, 2, ⋯, *n*) in Eq (7) can be computed based on the reliability estimation of complete data. We have

$$R(t_{i})=1-(i-0.5)/n$$ (11)

According to Eq (7), we can obtain the array column ((*t*_1_, *F*(*t*_1_)), (*t*_2_, *F*(*t*_2_)), ⋯, (*t*_*n*_, *F*(*t*_*n*_))). It is converted to a new array column ((*x*_1_, *y*_1_), (*x*_2_, *y*_2_), ⋯, (*x*_*n*_, *y*_*n*_)) by using Eqs (8) and (9). The sums of squared deviations are defined as

$$Q={\sum_{i=1}^{n}\left(y_{i}-\beta (t_{i}-\text{ln}\alpha )\right)}^{2}$$ (12)

The aim of the least square method is to find the estimated values of *α* and *β* that can minimize the sums of squared deviations. Specifically, we need to take the partial derivatives of parameters *α* and *β* and set the partial derivatives to 0. Then, we can get

$$\beta =\frac{L_{ty}}{L_{tt}}=\frac{\sum_{i=1}^{n}\left(t_{i}-\bar{t})(y_{i}-\bar{y}\right)}{\sum_{i=1}^{n}{\left(t_{i}-\bar{t}\right)}^{2}}$$ (13)

$$\alpha =\text{exp}(\bar{t}-\bar{y}/\beta )$$ (14)

In Eqs (13) and (14), the parameters $\bar{t}$ and $\bar{y}$ can be calculated by solving $\bar{t}=\frac{1}{n}\sum_{i=1}^{n}t_{i}$ and $\bar{y}=\frac{1}{n}\sum_{i=1}^{n}y_{i}$.

**Fig 4:**
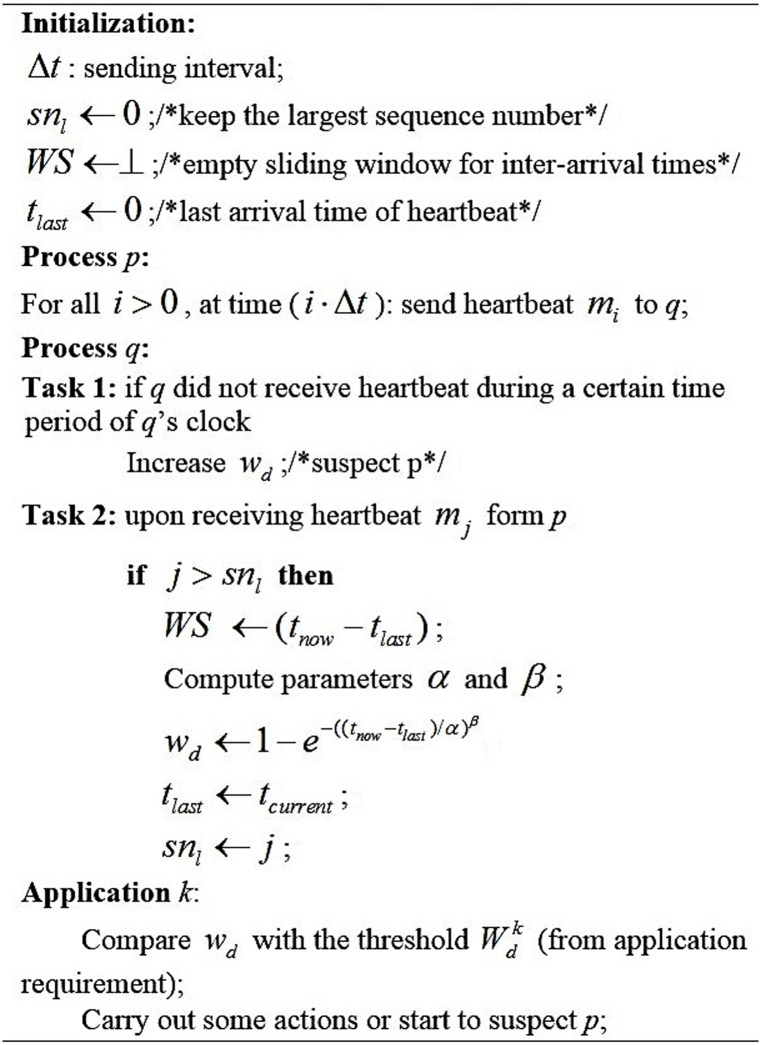
WD-FD algorithm.

As an accrual FD, the method used in WD-FD is quite simple. After the warm-up period, when a new heartbeat arrives, the inter-arrival time is put into a sliding window; simultaneously, the former oldest one is pushed out of the sliding window. Afterwards, the arrival time in the sliding window is used to calculate the parameters *α* and *β* of the Weibull distribution. Then, based on Eqs (5) and (6), we can calculate the current value of *w*_*d*_. Eventually, applications will compare the *w*_*d*_ value with its threshold *W*_*d*_; then, they will carry out some actions or start to suspect the process. Detailed information regarding the implementation of WD-FD is shown in Fig 4.

WD-FD is unable to get the communication delay from the sender to the receiver when it is lost. To ensure the effectiveness of the proposed approach, considering the influence of the message loss, we use the time theory to fill in the gap. Specifically, we fill in the gaps with a value computed by solving *d*_*i*_ = (Δ ⋅ *h*_*i*_) + *d*_*i*−1_, where Δ represents the average inter-arrival time in the sliding window and *h*_*i*_ means the average number of observed adjacent gaps.

### Correctness proof

From the theory point-of-view, the WD-FD can satisfy the accruement property and upper bound property. Therefore, our FD belongs to class *◇P*_*ac*_, which is sufficient to solve the consensus problem. The WD-FD implements a FD of class *◇P*_*ac*_ under the condition of the system model defined in section iii. The evidence is as follows.

If process *p* is faulty, the most recent arrival time of heartbeat *t*_*last*_ is constant; at time slot *t*_*k*_, the suspicion level *w*_*d*_ will be

$$sl_{qp}(t_{k})=w_{d}(t_{k})=1-e^{-{\left(\left(t_{k}-t_{last}\right)/\alpha \right)}^{\beta }}$$ (15)

As time passes, in time slot *t*_*k*+1_, the suspicion level is

$$sl_{qp}(t_{k+1})=w_{d}(t_{k+1})=1-e^{-{\left(\left(t_{k+1}-t_{last}\right)/\alpha \right)}^{\beta }}$$ (16)

Because *t*_*k*_ < *t*_*k*+1_, we get

$$-{\left(\left(t_{k+1}-t_{last}\right)/\alpha \right)}^{\beta }\leq -{\left(\left(t_{k}-t_{last}\right)/\alpha \right)}^{\beta }$$ (17)

$$e^{-{\left(\left(t_{k+1}-t_{last}\right)/\alpha \right)}^{\beta }}\leq e^{-{\left(\left(t_{k}-t_{last}\right)/\alpha \right)}^{\beta }}$$ (18)

Accordingly,

$$1-e^{-{\left(\left(t_{k+1}-t_{last}\right)/\alpha \right)}^{\beta }}\geq 1-e^{-{\left(\left(t_{k}-t_{last}\right)/\alpha \right)}^{\beta }}$$ (19)

This means that

$$sl_{qp}(t_{k+1})\geq sl_{qp}(t_{k})$$ (20)

At time slot *t*_*k*+*Q*_, *Q* > 0, *t*_*k*+*Q*_ > *t*_*k*_, using the above method and conclusion, we can get

$$sl_{qp}(t_{k+Q})>sl_{qp}(t_{k})$$ (21)

Therefore, the WD-FD satisfies the accruement property. Next, we continue to prove that the WD-FD satisfies the upper bound property.

If process *p* is correct, based on the system model, process *p* always makes progress in finite steps after some global time GST, i.e., *q* eventually receives the heartbeat from *p*. In other words, there exists *t*_*max*_ when the heartbeat from *p* arrives at *q*. At any arbitrary time *t*, where *t* ≤ *t*_*max*_,

$$sl_{qp}(t_{max})=w_{d}(t_{max})=1-e^{-{\left(\left(t_{max}-t_{last}\right)/\alpha \right)}^{\beta }}$$ (22)

$$sl_{qp}(t)=w_{d}(t)=1-e^{-{\left(\left(t-t_{last}\right)/\alpha \right)}^{\beta }}$$ (23)

Based on the accruement property, we know that *sl*_*qp*_(*t*) ≤ *sl*_*qp*_(*t*_*max*_) = *SL*_*max*_. Thus, the WD-FD satisfies the upper bound property.

## Performance evaluation

To evaluate and analyze the performance of WD-FD, we choose similar *φ* FD and ED FD for comparative experiments, both of which are accrual FDs. To increase the authenticity of the comparative experiments, we first used classical experimental data obtained from a WAN environment and applied *φ* FD and ED FD. The experimental data were obtained in a WAN environment. Furthermore, we rented the cloud services of Amazon and built the experimental platform to present the performance of WD-FD. We referred to the method in paper [2], i.e., making use of the same trace files to replay the different schemes of FDs, and calculated the QoS metrics. This method could ensure that all of the schemes of FDs are compared under the same network conditions.

For the WAN scenario, the experiment involved two computers: one located in Japan and the other in Switzerland. The two computers communicated through a normal intercontinental Internet connection. One machine was responsible for sending heartbeats while the other for recording the arrival time of each heartbeat. Neither machine failed during the experiment. The heartbeats were sent with a target of one heartbeat every 103.501 ms (standard deviation: 0.19 ms; min.: 101.7 ms; max.: 234.3 ms). During the experiment, the round-trip time (RTT) was measured to be at a low rate. The average RTT was 283.3 ms, with a standard deviation of 207.3 ms, minimum of 270.2 ms, and maximum of 717.8 ms. More than 5 million heartbeats were received, and the loss rate was approximately 0.4%.

**Fig 5:**
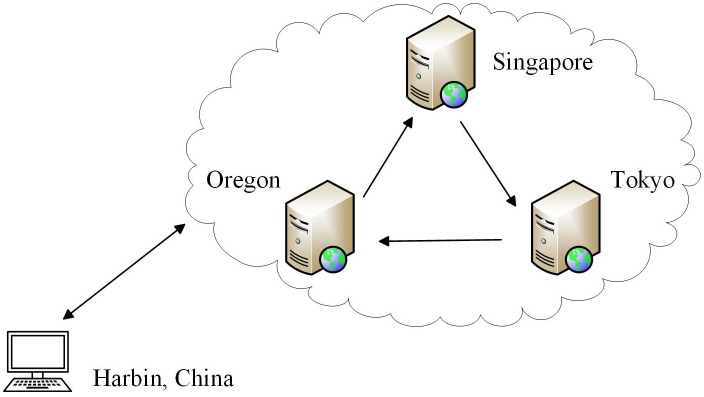
The experimental environment of cloud computing.

For the cloud computing scenario, an experimental platform was built by renting the Amazon EC2. Some servers located in Tokyo, Singapore, and Oregon (USA) were selected to provide a query service based on the Web. These servers were equipped with a 2.5 GHz Intel Xeon processor, 1 GHz of memory and the Red Hat Linux 7.2 operating system. We assumed that the client had already known the network address of the three servers. The client in Harbin (China) connected the server first in Oregon and then in Singapore and Tokyo; the service was stopped via the fault injection method. The experimental environment is shown in Fig 5. The sending interval was set to 2 s, while the measured sending rate was actually one heartbeat every 2.092 s (standard deviation: 0.019 s; min.: 1.964 s; max.: 5.239 s). During the experiment, the average RTT was 0.1792 s, with a standard deviation of 0.0086 s, minimum of 0.1187 s, and maximum of 14.505 s. More than 3 million heartbeats were received, with a loss rate of approximately 0.72%.

In the experiments, each FD scheme applied a sliding window to save past samples to compute their future suspicion levels. All of the experiments for the three FDs shared the same fixed window size (*WS* = 1000). Furthermore, we did not analyze the sampled data until the sliding window was full, as the behavior of the FDs is stable only after that moment.

The parameters of FDs are configured as follows: to find the best QoS and compare with the others, here, *E*_*d*_ ∈ [10^−4^,10] for ED FD, as in [15]; for *φ* FD, the parameters are the same as those in [14]: Φ ∈ [0.5, 16]; *W*_*d*_ ∈ [0, 1] for WD-FD.

In the experiments, the mistake rate, detection time and query accuracy probability were selected as the key performance metrics. Different values of these metrics were obtained in each experiment based on the respective parameters.

In all experiments, we calculate an estimation for average detection time *T*_*D*_ as follows. Assuming that a crash would occur exactly after successfully sending a heartbeat (worst-case scenario), we measure the time elapsed until the FD would report a suspicion for each analyzed sample. With *φ*, ED and WD FDs, we consider the algorithms’ threshold values (Φ, *E*_*d*_ and *W*_*d*_) and reverse the computation of *φ*, *e*_*d*_ and *w*_*d*_ to obtain the equivalent timeout each time a new heartbeat is received and take the mean value Δ_*to*_. We estimate the mean propagation time Δ_*tr*_ based on RTT. Then, for each sample, we compute the average (worst-case) detection time as follows.

$$T_{D}\approx \Delta_{to}+\Delta_{tr}$$ (24)

### Experiment in a WAN

**Fig 6:**
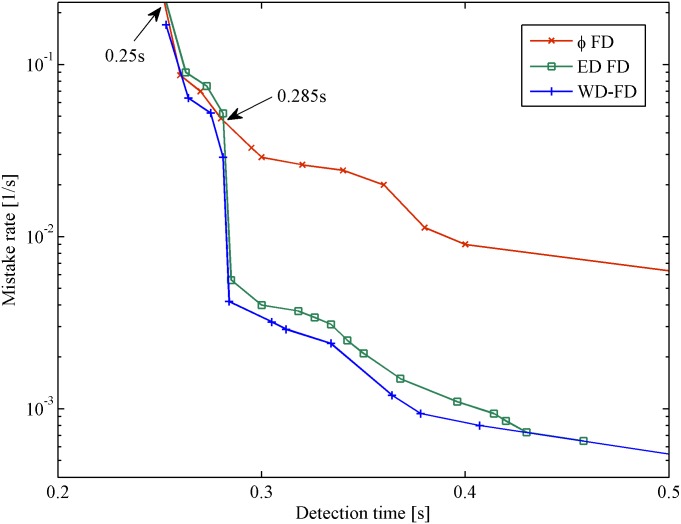
Mistake rate vs. detection time in WAN.

**Fig 7:**
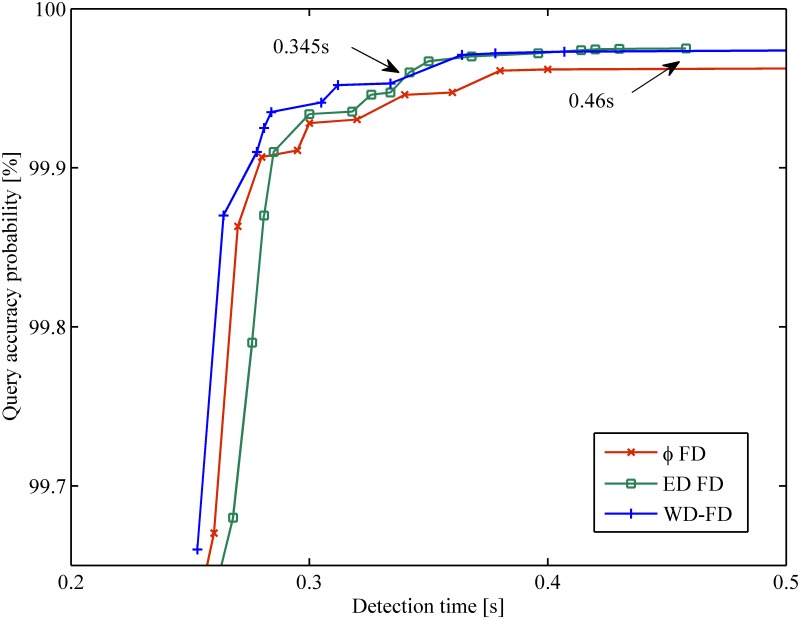
Query accuracy probability vs. detection time in WAN.

Fig 6 shows the results of the mistake rate *λ*_*M*_ vs. detection time *T*_*D*_ in the WAN scenario. The x-coordinate represents the detection time, and the y-coordinate represents the mistake rate. Fig 7 shows the results of query accuracy probability *P*_*A*_ vs. detection time *T*_*D*_ in the same scenario.

From the figures, we found that all of the FDs follow the same general tendency. However, our FD outperforms the others in the WAN scenario. This improvement is because most late heartbeats were caught by the corresponding thresholds under the same network conditions. In Fig 6, when 0.25*s* < *T*_*D*_ < 0.285*s*, *φ* FD has a lower mistake rate than ED FD. In the WAN scenario, losing a single heartbeat is considered a normal situation [14]. The lost heartbeats influenced the calculation of the timeout value Δ_*to*_. More heartbeats were caught by *φ* FD; thus, it has a lower mistake rate than ED FD during that period. The Weibull distribution can be converted to an exponential distribution when the parameter *β* = 1. Moreover, the Weibull distribution is similar to the normal distribution when the parameter *β* > 1. Thus, our proposed FD can catch more heartbeats than other FDs such that the mistake rate is reduced. From Fig 7, when *T*_*D*_ < 0.345*s*, our FD has higher query accuracy probability than the others. With increasing detection time, our FD and ED FD have similar query accuracy probability when 0.345*s* < *T*_*D*_ < 0.46*s*. In the aggressive range (*T*_*D*_ < 0.5*s*), our FD presents the lowest mistake rate (an improvement up to 20%), as well as the best query accuracy probability for the most measured detection time.

### Experiment in cloud computing

**Fig 8:**
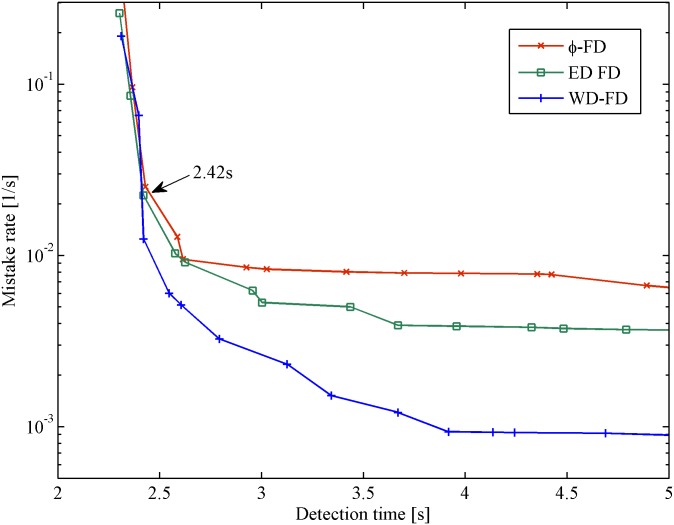
Mistake rate vs. detection time in cloud computing.

**Fig 9:**
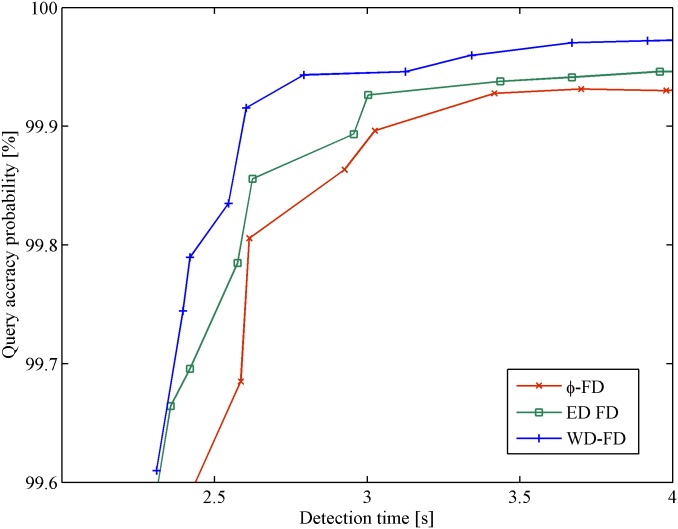
Query accuracy probability vs. detection time in cloud computing.

Figs 8 and 9 show the results of the mistake rate *λ*_*M*_ and query accuracy probability *P*_*A*_ vs. detection time *T*_*D*_ in the cloud computing scenario. Similar to the result in the WAN scenario, the mistake rate and query accuracy probability of all of the FDs have an identical tendency with increasing detection time. In Fig 8, *φ* FD and ED FD turn earlier compared with WD-FD. This is because the heartbeats loss tends to occur during the period of switching servers. In such a network condition, WD-FD can catch more heartbeats than the others. It presents the lowest mistake rate (an improvement of up to 80%) when *T*_*D*_ > 2.42*s*. In Fig 9, our FD has an obvious improvement (approximately 6%) compared with ED FD. Furthermore, when the mistake rate or query accuracy probability is the same, our FD has a shorter detection time. In cloud computing, WD-FD behaves better than the other FDs in terms of low mistake rate, short detection time and high query accuracy probability.

From all of the above results, the WD-FD presents the best performance in scenarios of unstable network conditions (especially in cloud computing) when compared to the most relevant existing algorithms for failure detection. WD-FD is an effective improvement over ED FD and *φ* FD in terms of low mistake rate, short detection time and high query accuracy probability.

## Conclusion

Failure detection plays a very important role in dependable distributed systems. In this paper, we introduced the WD-FD based on the Weibull distribution. It has been proven that WD-FD is an accrual FD of class *◇P*_*ac*_. By using the Weibull distribution to estimate the distribution of heartbeat inter-arrival time, the WD-FD can adapt well to changing network conditions (especially the cloud service environment) and the requirements of any number of concurrently running applications. Moreover, the information of processes’ failures is useful to guide service conditions and cloud resources in cloud computing. Through comparative experiments, the results showed that WD-FD demonstrates a better performance in terms of false detections when compared to existing accrual FDs in cloud computing. Therefore, WD-FD is a suitable layout in cloud computing for providing the failure detection service.

## Supporting information

S1 File Cloud.zip. (ZIP)

S2 File Wan.zip. (ZIP)
